# Deep-Learning-Based Wireless Visual Sensor System for Shiitake Mushroom Sorting

**DOI:** 10.3390/s22124606

**Published:** 2022-06-18

**Authors:** Junwen Deng, Yuhang Liu, Xinqing Xiao

**Affiliations:** College of Engineering, China Agricultural University, Beijing 100083, China; d2907666256@163.com (J.D.); 2019307160224@cau.edu.cn (Y.L.)

**Keywords:** mushroom sorting, deep learning, wireless sensor

## Abstract

The shiitake mushroom is the second-largest edible mushroom in the world, with a high nutritional and medicinal value. The surface texture of shiitake mushrooms can be quite different due to different growing environments, consequently leading to fluctuating market prices. To maximize the economic profit of the mushroom industry, it is necessary to sort the harvested mushrooms according to their qualities. This paper aimed to develop a deep-learning-based wireless visual sensor system for shiitake mushroom sorting, in which the visual detection was realized by the collection of images and cooperative transmission with the help of visual sensors and Wi-Fi modules, respectively. The model training process was achieved using Vision Transformer, then three data-augmentation methods, which were Random Erasing, RandAugment, and Label Smoothing, were applied under the premise of a small sample dataset. The training result of the final model turned out nearly perfect, with an accuracy rate reaching 99.2%. Meanwhile, the actual mushroom-sorting work using the developed system obtained an accuracy of 98.53%, with an 8.7 ms processing time for every single image. The results showed that the system could efficiently complete the sorting of shiitake mushrooms with a stable and high accuracy. In addition, the system could be extended for other sorting tasks based on visual features. It is also possible to combine binocular vision and multisensor technology with the current system to deal with sorting work that requires a higher accuracy and minor feature identification.

## 1. Introduction

**Figure 1:**
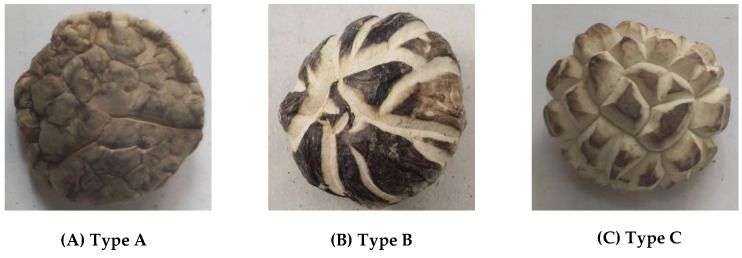
Sorting of shiitake mushrooms according to texture.

The shiitake mushroom is the second-largest edible mushroom in the world. It has a fresh taste and tender texture, and is of high nutritional and medicinal value [1,2]. During the growth of shiitake mushrooms, the relatively fast-growing fleshy cells can burst the epidermal layers, causing the cap to crack, thus forming a brown and white pattern on the top [3,4,5]. External environment factors also play an essential role in the surface texture of the cap of the mushrooms, such as light, temperature, and pH value [6]. As shown in Figure 1, according to the surface texture of the cap, shiitake mushrooms can be classified as smooth mushrooms, which appear with no texture on the surface of the cap, as shown in Figure 1A; camellia mushrooms, which appear with scattered cracks on the surface of the cap, as shown in Figure 1B; and white mushrooms, which appear with exaggerated cracks and a star-shaped black spot on the cap, as shown in Figure 1C. Mushrooms with different textures have different market values [7]. Compared with common smooth mushrooms (e.g., Figure 1A), the market price of white mushrooms (e.g., Figure 1C) can reach 5 to 8 times that of smooth mushrooms. Therefore, to maximize the profit of the mushroom industry, it is necessary to sort the mushrooms in line with their cap textures.

Contrary to the current trend in automated production, the sorting process of shiitake mushrooms is still mainly carried out manually, leading to high work intensity and low sorting efficiency. With the development of agricultural science and intelligent detection technology, machine vision technology was introduced to identify and classify agricultural products [8,9]. The identification efficiency could be effectively improved since related images could be collected through cameras, and image-processing algorithms were applied to analyze the images. Additionally, machine vision avoided the subjective deviation caused by the emotional influence of the human during detection. Machines surpass humans in terms of workload, efficiency, and correctness. However, the image-processing algorithms used in machine vision are confined to relatively simple features and specific targets, such as the Canny algorithm for edge detection and Hough Transform for geometric figure detection. The textures of mushrooms, considering their randomness and abstraction affected by the natural environment, were still too difficult for the image-processing algorithm to identify accurately.

Compared with traditional machine vision and image-processing algorithms, deep learning technology has significant advantages over conventional machine vision and image-processing algorithms in image identification, in which the subjects are more abstract and complex, and features are difficult to extract directly [10]. To analyze a given target with complex features, it only requires a training set consisting of a certain number of pictures and their labels with appropriate parameters. The deep learning model can then automatically extract features and carry out model training. As a result, the workload of algorithm design is significantly reduced [11].

Vision Transformer (ViT) is a network model that introduces the self-attention mechanism into vision. After pretraining with a large amount of data and transferring the pre-training model to small and medium datasets, ViT is more robust than convolutional neural networks (CNNs). The required computing resources could be significantly reduced, as well as the risk of overfitting in small and medium datasets [12,13]. Transformers are gradually becoming a popular approach in computer vision fields such as image classification, image segmentation, and object detection. In this paper, a set of shiitake mushroom texture images were collected through a visual sensor for training the ViT model, and finally obtained a ViT model suitable for shiitake mushroom texture sorting.

Subjected to the limited space in the real-world production environment, transmitting the collected mushroom images wirelessly is more than necessary. Along with high transmission efficiency, wide coverage, and low transmission power, Wi-Fi technology stands out for its incredible speed and stability in terms of image transmission [14]. Using wireless transmission technology to transmit the collected mushroom images ensures the flexibility of other working machines in the production space and facilitates the supervision of the entire mushroom sorting system.

As described in the above discussion, the main objective of this study was to design a deep-learning-based wireless visual sensor system for shiitake mushroom sorting. The visual detection was realized by using collecting images and cooperative transmission with the aid of visual sensors and Wi-Fi modules, respectively. The training results of the ViT model and the shiitake mushroom sorting performance of the entire system were comprehensively analyzed and discussed. This developed deep-learning-based wireless visual sensor system could effectively detect the surface texture of mushrooms in real time and transmit the image to the remote terminal. The sorting of different types of mushrooms was finally completed after the image was judged by the trained model, improving the economic benefits for the mushroom industry.

The rest of this paper is organized as follows. The deep-learning-based wireless visual sensor system’s materials and methods are discussed in Section 2. The performance evaluation results of the deep-learning-based wireless visual sensor system are demonstrated in Section 3. Finally, the conclusions and future work are presented in Section 4.

## 2. Materials and Methods

The materials and methods involved in this paper, including the design of the wireless visual sensor system, the training method of the deep learning model, and the experimental scheme of the actual mushroom sorting, are presented with more detail in this section.

### 2.1. Wireless Visual Sensor System

**Figure 2:**
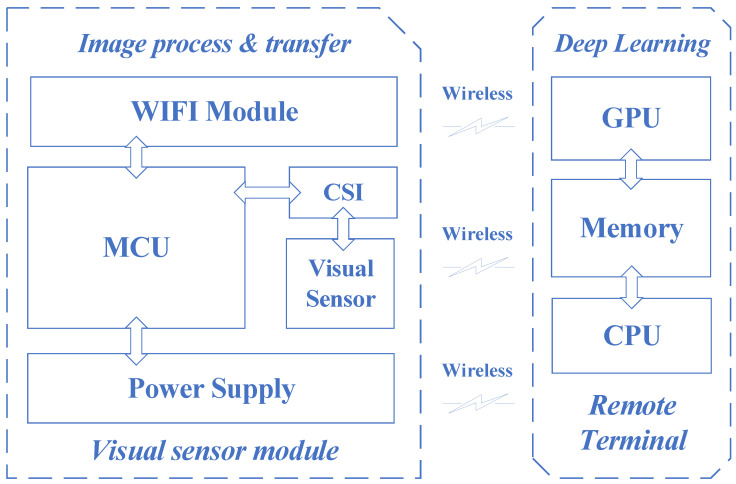
Block diagram of deep-learning-based wireless visual sensor system.

**Figure 3:**
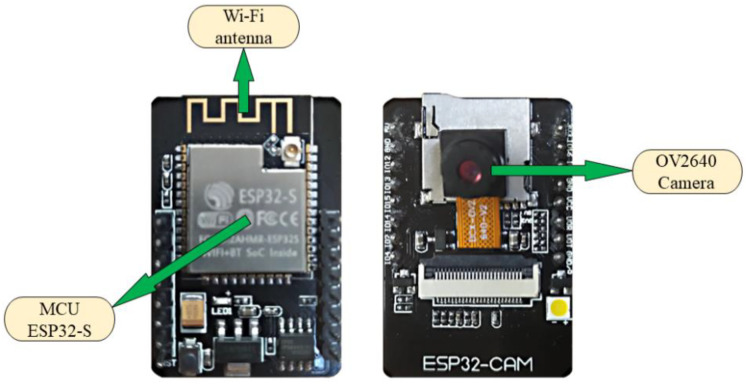
Physical implementation of deep-learning-based wireless visual sensor system.

The wireless visual sensor system consisted of a visual sensing module and a remote terminal. The visual sensor module, as shown in Figure 2 and Figure 3, included a visual sensor (DCX-OV2640-V2, ALIENTEK, Guangzhou, China) and a microcontroller unit (ESP32-S, ANXINKE, Shenzhen, China) with an integrated Wi-Fi module. The image resolution of the visual sensor was 1024 × 768. The remote console consisted of a CPU (Intel Xeon Gold 6240 2.6 GHz), GPU (NVIDIA Tesla V100 32 GB),and internal memory.

Apart from adjusting the sampling interval of the vision sensor and coordinating and transmitting images, the microcontroller unit could also perform simple image processing on the collected mushroom texture images to achieve image denoising and optimize the region of interest. The remote terminal processed the transmitted images through the deep learning algorithm and then fed back the results to the microcontroller unit for subsequent packaging of the mushrooms.

The microcontroller unit used in this paper was powered by a 5V DC power supply. The visual sensor was connected to the control module through the complementary metal–oxide–semiconductor (CMOS) sensor interface (CSI,) while the Wi-Fi module was integrated into the chip. Both were powered by the voltage regulator power supply circuit in the microcontroller unit.

**Figure 4:**
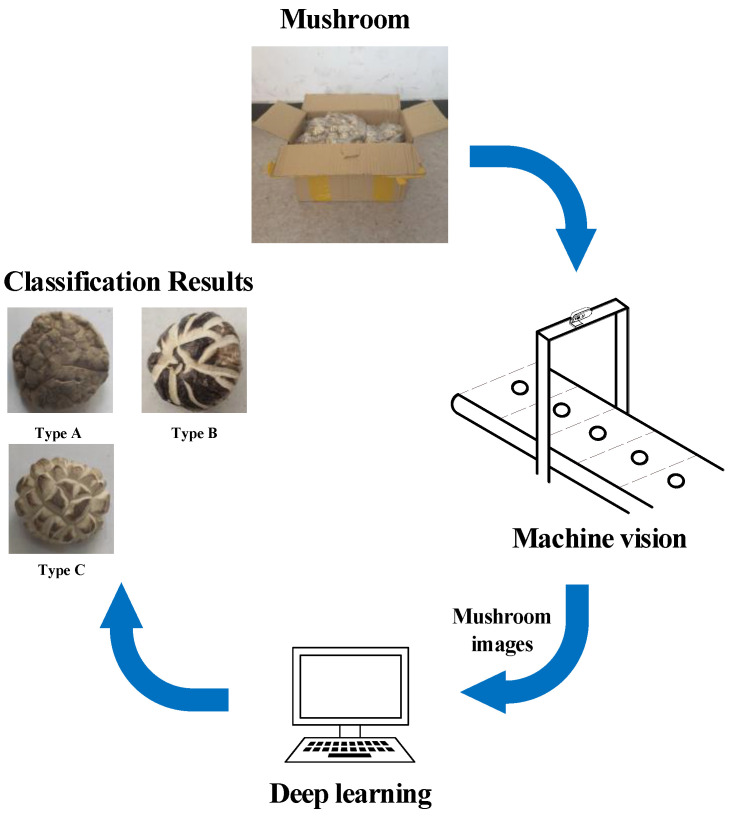
Workflow of deep-learning-based wireless visual sensor system.

The workflow of the deep-learning-based wireless visual sensor system is shown in Figure 4. The harvested shiitake mushrooms were arranged one by one on the conveyor belt, which moved at a speed of 0.3 m/s at a distance of 0.3 m. The visual sensing module equipped with a camera was fixed on the bracket of the conveyor belt and placed above the mushroom to be detected. The distance between the camera and the sampling plane of the mushroom was 0.3 m. Once the microcontroller unit received the signal from the remote terminal, the camera began collecting mushroom images. The microcontroller unit would then perform simple image-preprocessing schemes (denoising and cropping) and transmit the processed image to the remote terminal via the Wi-Fi module. Afterward, the remote terminal classified the collected texture images of the shiitake mushrooms based on the deep learning model, and finally sent the sorting results back to the microcontroller unit for subsequent packaging of the shiitake mushrooms. The process was conducted using the following machinery on the assembly line.

### 2.2. Obtaining Datasets and Preprocessing Images

To train the model, we manually selected 100 shiitake mushrooms from each of the three types, resulting in a total of 301 samples. Each sample was defined and labeled by three different expert farmers from an agricultural cooperative, and the results followed the majority rule. Three images of each sample were taken in different illuminations and angles to rule out irrelevant features that could result in discrimination. A total of 903 images of mushrooms were obtained in the end for model training; 80% of these images were used as training sets, while the other 20% were used as the validation set. The numbers of images of the three mushroom types were the same in the training set.

After the images were obtained, several methods were used to preprocess them. A Gaussian filter with a 3 × 3 kernel was firstly used for image denoising. Then, all the images were cropped to the same size to set the region of interest. After that, they were resized to 224 × 224 before being sent to the remote terminal for neural network training.

### 2.3. Augmentation and Regularization

Data augmentation is widely used in neural network training. A large amount of sample data is often required to train a neural network. Still, due to instrumental limitations, a dataset with fewer samples (3 classes, a total of 903 images) was used for training. In addition, the small number of samples obtained by manual sorting was expected to allow the machine to learn how to complete the subsequent large-scale sorting.

However, with inadequate training samples, a deep learning model will be more likely to overfit, making it difficult to obtain meaningful training results. Moreover, the weaker inductive bias of Vision Transformer often leads to increased reliance on data augmentation or model regularization when trained on smaller training datasets com-pared to convolutional neural networks [15]. Therefore, a transfer-learning strategy was adopted, and three methods of augmentation and regularization were applied to the dataset, which were Random Erasing, RandAugment and Label Smoothing.

#### 2.3.1. Random Erasing

**Figure 5:**
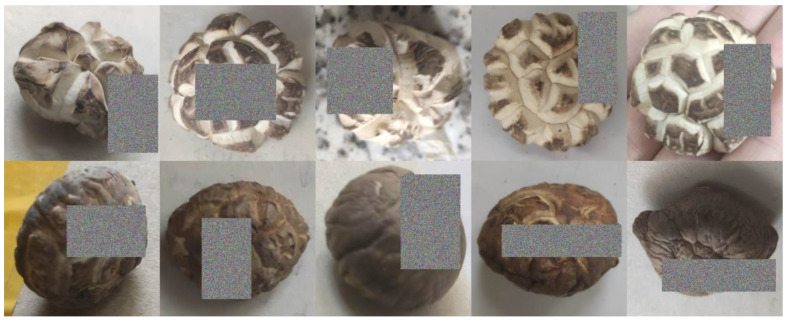
Shiitake mushroom images after Random Erasing.

Random Erasing is a data augmentation method used in visual machine learning. Applying this method in data augmentation can effectively reduce the risk of overfitting by a neural network model. As shown in Figure 5, during the training process, the input samples had a certain probability of undergoing the following processing: a rectangular area of any size was randomly selected in the picture, in which the pixels were covered with random values or average values. After the process, a series of augmented images could be obtained with various occlusion levels [16]. Using this data-augmentation method could force the neural network model to learn a broader range of features in the sample and enable the trained model to better cope with scenes in which part of the mushroom texture was covered, thereby improving the generalization ability and robustness of the model. At the same time, this method was also a supplement to the previous data-enhancement methods (such as flipping, shearing, or translation, etc.).

#### 2.3.2. RandAugment

To further reduce the risk of overfitting and improve the training effect, we also employ RandAugment in the training. RandAugment is an automatic data-augmentation strategy. Compared with previous automatic data-augmentation methods, it introduces a minimal search space with only two interpretable hyperparameters, effectively reducing the training complexity and computational expense. The available data augmentation methods included in RandAugment (K=14) are:

• identity   • autoContrast   • equalize  • rotate

• solarize   • color     • posterize    • contrast

• brightness • sharpness      • shear-x    • shear-y

• translate-x • translate-y

According to the size of the model and dataset, RandAugment randomly selects N types of data-augmentation methods from the above for model training, where each method has an equal probability of being selected, meaning there are C(K,N) potential augmentation strategies. At the same time, a hyperparameter M is introduced to control the strength of image data distortion in each method [17]. Since there are only two hyperparameters, RandAugment uses a naïve grid search, which significantly reduces its search space, and can obtain multiangle augmented samples with only a small computational cost. RandAugment can instantly be used on different tasks and datasets with a high working efficiency thanks to these properties.

#### 2.3.3. Label Smoothing

Label Smoothing is a regularization strategy that adds noise through soft one-hot to reduce the weight of the actual sample label category when calculating the loss function [18], as given in Equation (1):

(1) $$y_{i}=\{\begin{array}{c} 1,\;i=target\\ 0,\;i\neq target \end{array}\;\;\;\;\;\;\;\;\;\;\;\;\Rightarrow \;\;\;\;\;\;\;\;\;\;\;\hat{y_{i}}=\{\begin{array}{c} 1-\alpha ,\;i=target\\ \alpha /K,\;i\neq target \end{array}$$

where $y_{i}$ is the sample label before and after, α is the label smoothing factor, and K is the number of categories.

Using Label Smoothing in training can effectively suppress the overfitting problem, enhance the model’s generalization ability, and improve the model’s predictive ability in practical application. In addition, biased manual sorting can sometimes lead to confusion. For example, some samples own both features of type A and type B mushrooms; Label Smoothing could easily overcome this dilemma and prevent poor generalization performance.

### 2.4. Vision Transformer (ViT)

**Figure 6:**
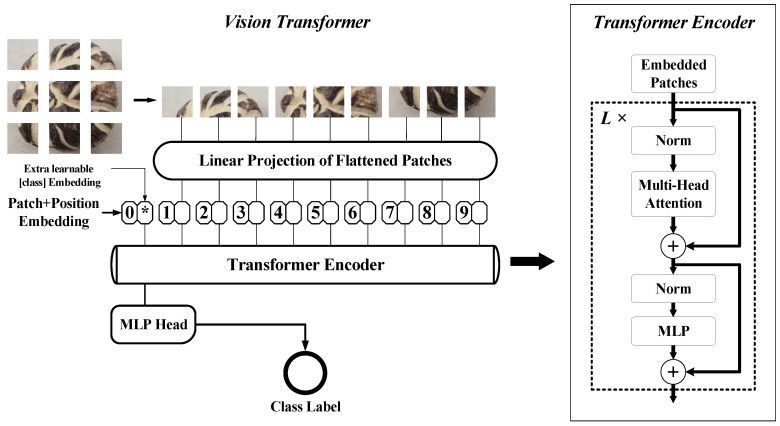
Structure of Vision Transformer.

ViT is an image-classification model based on a pure transformer structure that, along with its variants, is gradually being applied in various fields. The core process of ViT includes creating patches, patch embedding, positional encoding, transformer encoding, and multilayer perceptron (MLP) classification [19], as shown in Figure 6.

The input of ViT is typically an RGB three-channel image. An image $x\in R^{H\times W\times C}$ (H: height, W: width, C: channels) was firstly be divided into blocks, then reshaped into a sequence of flattened 2D patches $x_{p}\in R^{N\times \left(P^{2}\cdot C\right)}$, where P×P×C is the size of each patch and $N=HW/P^{2}$ was the resulting number of patches. The patches were then flattened and mapped into vectors of size D using a trainable linear projection as patch embeddings.

It was noteworthy that before these vectors were input into the transformer encoder, a trainable (class) token was required for classification, and a position embedding was needed to preserve the position information in the image. The obtained series of vectors were then used as the input of the transformer encoder. The transformer encoder mainly included two parts: MSA (multihead self-attention) and MLP. The forward process can be summarized as follows:

(2) $$z_{0}=\left[x_{class};x_{p}^{1}E;x_{p}^{1}E;\cdots ;x_{p}^{N}E\right]+E_{pos}\;\;\;\;\;\;\;\;\;\;\;\;E\in R^{\left(p^{2}\cdot C\right)\times D},E_{pos}\in R^{\left(N+1\right)\times D}$$

(3) $$z_{l}^{\prime }=MSA\left(LN\left(z_{l-1}\right)\right)+z_{l-1}\;\;\;\;\;\;\;\;\;\;\;\;\;\;\;\;\;\;\;\;\;\;\;\;\;\;\;\;\;\;\;\;\;\;\;\;l=1\cdots L$$

(4) $$z_{l}=MLP\left(LN\left(z_{l}^{\prime }\right)\right)+z_{l}^{\prime }\;\;\;\;\;\;\;\;\;\;\;\;\;\;\;\;\;\;\;\;\;\;\;\;\;\;\;\;\;\;\;\;\;\;\;\;\;\;\;\;\;\;\;l=1\cdots L$$

(5) $$y=LN\left(z_{L}^{0}\right)\;\;\;\;\;\;\;\;\;\;\;\;\;\;\;\;\;\;\;\;\;\;\;\;\;\;\;\;\;\;\;\;\;\;\;\;\;\;\;\;\;\;\;\;\;\;\;\;\;\;\;\;\;\;\;\;\;\;\;\;\;\;\;\;\;\;\;\;\;\;\;\;\;\;\;\;\;\;\;\;\;\;$$

As is shown in Equation (2), $x_{p}^{1}$ to $x_{p}^{N}$ stands for N flattened 2D patches, and E represents the fully connected layer of linear projection, which is used to obtain patch embeddings. $x_{class}$ is the trainable (class) token additionally added to obtain the final output of classification. $z_{0}$ is finally obtained as the input of the transformer encoder after adding position-embedding $E_{pos}$ to preserve position information in the image. The following process contains totally L repeated operations of MSA and MLP. As were shown in Equations (3) and (4), $z_{l}^{\prime }$ and $z_{l}$ represent the result of MSA and MLP after one operation, respectively, LN is the layer norm that all embedded patches had to go through before the MSA and MLP operations. Ultimately, the state of $x_{class}$ at the output of the transformer encoder ($z_{L}^{0}$) served as the image representation (y). The final classification result was obtained by attaching the MLP head to the image representation.

The commonly used ViT models are of 5 different sizes, classified by the number of embedding dimensions and the number of parameters. The processing speed and accuracy will differ due to different sizes, so that they can be applied to different problems. Generally, there are five different sizes of models available, which are Tiny, Small, Base, Large, and Huge. Their parameter quantities are 6M, 22M, 86M, 307M, and 632M, respectively. In addition, models of the same size also vary by the size of patches into which an input image is divided; 8×8, 14×14, 16×16, and 32×32 are the typical sizes of patches. This article used the model size and patch size to describe a specific ViT model. For example, ViT-Base/16 is a ViT model with a size of Base and a patch size of 16×16. Only Tiny-, Small-, and Base-sized models were used in the experiments, as the computational cost of larger models would become unbearable.

### 2.5. Model Training Methods

All the experiments in this study were carried out on a computer equipped with an Intel(R) Xeon(R) Gold 6240 CPU @ 2.60 GHz, an NVIDIA Tesla V100 32 GB GPU, and 32 GB of RAM. The software environments in this study were Python 3.9, Torch 1.11.0, and CUDA 11.0. The operating system was Linux.

Considering the actual production and application situation, we adopted the transfer-learning method to reduce the time cost of training. The models in this paper were pretrained on the public dataset ImageNet-21k (14 million images; 21,843 classes).

During the experiment, 40 epochs were set for the finetuning of the model with a batch size of 64. Adam was used as the optimizer for the gradient descent calculation, and the learning rate was set to $1\times 10^{-3}$, with 10 epochs set to the warmup learning rate. After training for 30 epochs, we decayed the learning rate to 0.1 of its original one. The probability taken by Random Erasing during the training process was 0.5, which meant 50% of the input images went through Random Erasing. The label-smoothing factor *α* was set to 0.1.

To evaluate the progress of our model training, we applied the cross-entropy loss function, which was the measurement of the difference between two probability distributions. It has been widely used in the field of machine learning and deep learning [20]. In model training, the cross-entropy loss function was used to represent the closeness of the predicted data distribution to the actual data distribution, as defined below in Equation (6):

(6) $$Loss=\frac{1}{N}\underset{i}{\sum }L_{i}=-\frac{1}{N}\underset{i}{\sum }\overset{M}{\underset{c=1}{\sum }}y_{ic}\log\left(p_{ic}\right)$$

where M represents the number of categories, and $y_{ic}$ is the sample’s label. If the true category of sample i is equal to c before using label smoothing, $y_{ic}$ equals 1; otherwise, it equals 0. $p_{ic}$ represents the predicted probability that the observed sample i belongs to category c.

### 2.6. Experimental Scheme

The experiment materials, shiitake mushrooms with no mechanical damage, were harvested at an agricultural cooperative in Xixia, Henan, central-eastern China. All three types of shiitake mushrooms, including smooth mushrooms, white mushrooms, and camellia mushrooms, were manually sorted and packaged by the local farmers.

In the experiment, the camera was set to collect mushroom images at a 1 s interval. We applied ViT-Base/16 as our final model to sort the mushrooms. A total of 204 mushrooms were used to test the system’s performance in the experiment, and the sorting results were ultimately compared to those sorted manually to evaluate the result of the experiment. Moreover, the experiment was conducted multiple times. Before each experiment was repeated, the samples were disrupted and distributed randomly to each dataset so that we could determine whether the small number of samples would affect the reliability of our experiment results.

During this experiment, the training results of deep learning models, the accuracy of mushroom sorting, and the possibility of extended usage were analyzed to show the edge of our model. The performance in shiitake mushroom sorting was evaluated to ensure the efficiency of the entire sensor system. Finally, we engaged in further discussions to ensure the reliability of our experiment results.

### 2.7. Data Analysis

In terms of evaluating the effect of the model after training, the parameters of accuracy, positive predictive value, true positive rate, F1-score, and processing speed were introduced.

Acc (Accuracy) represented the proportion of correctly predicted samples in the test sample, as shown in Equation (7):

(7) $$Acc=\frac{n_{c}}{n_{t}}\times 100\%$$

where $n_{t}$ is the total number of samples tested, and $n_{c}$ is the number of correctly predicted samples.

The positive predictive value (PPV) represented the proportion of the samples predicted to be positive and was actually a positive sample, as shown in Equation (8):

(8) $$PPV=\frac{TP}{TP+FP}$$

where TP is the positive samples that were correctly predicted, and FP is the negative samples that were incorrectly predicted as positive samples.

The true positive rate (TPR) represented the proportion of correctly predicted positive samples, as shown in Equation (9):

(9) $$TPR=\frac{TP}{TP+FN}$$

where FN is the negative samples that were incorrectly predicted as positive samples.

Since PPV and TPR seldom achieve good results simultaneously, the F1-score was introduced to comprehensively measure the effects of PPV and TPR output, as shown in Equation (10):

(10) $$F1=2\frac{PPV\cdot TPR}{PPV+TPR}$$

Python 3.9 and Microsoft Office Excel 2021 were used for the calculation and statistics of the training result data.

## 3. Results and Discussions

According to the experimental scenario, the network training results, the performance in the shiitake mushroom sorting, and the experiment results’ reliability are analyzed and discussed in this section in more detail.

### 3.1. Network Training Results

**Figure 7:**
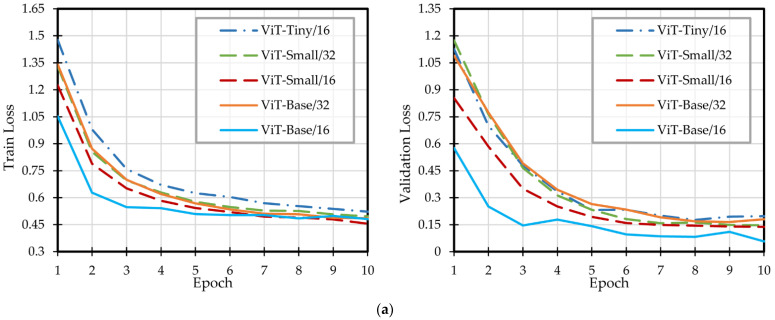

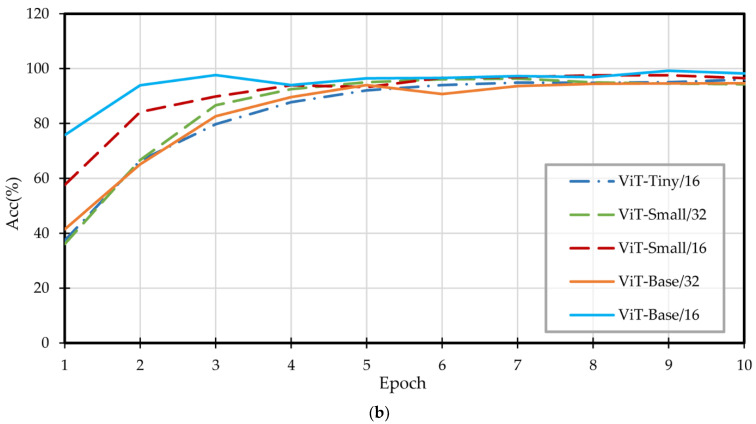
Training curves of ViT. (**a**) Loss curves of the training set and validation set; (**b**) training curves of the validation set.

During the model training process, the curves of the Loss value and Acc value of the validation set were obtained as two functions of epochs. Both curves went flat within 10 epochs, which explained the presentation of the Loss and Acc curves of 10 epochs in Figure 7. The best results obtained during training and the time spent in training are dis-played in Table 1.

As shown in Table 1 and Figure 7, when all models were under the same training conditions, ViT-Base/16 had the lowest Loss, reaching 0.057, and achieved the best Acc of 0.992, while relatively small models such as ViT-Small/16 took only 177 s to complete an Acc of 0.989.

The results showed that the ViT model had a stable and high accuracy in the texture identification of shiitake mushrooms. In addition, the Loss curve decreased rapidly during training and the training time was short, which could well meet the actual sorting needs.

### 3.2. Performance in Shiitake Mushroom Sorting

**Figure 8:**
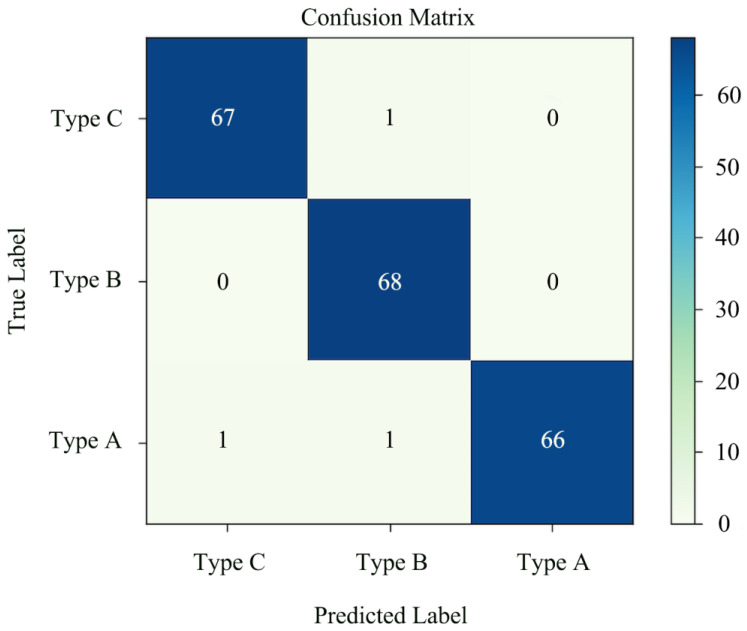
Confusion matrix of ViT-Base/16.

According to the experimental plan, the deep-learning-based wireless visual sensor system was used for shiitake mushroom sorting to sort the actually harvested mush-rooms. Here, we applied ViT-Base/16, and the results are shown in Figure 8 and Table 2. The same dataset and training strategy were also used to simultaneously test some common image-classification networks to obtain the results in shown Table 3.

As shown in Figure 8 and Table 2, the overall accuracy rate of the shiitake mushroom sorting was 98.53%. The resulting model did not mistakenly include other species in Type A. At the same time, all the given Type B samples were successfully detected correctly. The F1-scores of the three types were similarly high, which indicated that the model had a better judgment ability for each type.

As shown in Table 3, compared with the previous CNN model using the same trans-fer-learning strategy, the ViT model had an Acc of 99.2%, which was higher than that of the general CNN network. At the same time, its inference speed reached 6.8 ms/image, which was only half of the time when using a traditional CNN network. With high accuracy and efficiency, the training time of the ViT model did not become longer, which undoubtedly highlighted the advantages of the ViT model in the sorting tasks.

The results above showed that this deep-learning-based wireless visual sensor system could efficiently and accurately complete the work of shiitake mushroom sorting. It can undoubtedly ensure the production efficiency and economic benefits of the shiitake mushroom industry. When dealing with classification tasks with very few training samples, the ViT model performed better, with a faster inference speed and a higher accuracy. Therefore, the ViT network has good application prospects and value in similar production tasks of quality sorting.

### 3.3. Further Discussions of the Reliability of the Results

In the model training, a preselection of the mushrooms was achieved manually by the farmers. The preselection aimed to ensure that each sample in the dataset could represent a clear example of its type, so that our model would output results close to manual sorting. However, manual sorting is often subjective, and the training samples were likely to be biased. In that case, our system would provide convenience for the sorting work as long as our model output was consistent with manual sorting.

In addition, multiple experiments with the models showed that for all the ViT models, the Acc (%) floated within at most 1%, revealing convincing results despite the small scale of our test set.

## 4. Conclusions

This paper developed a deep-learning-based wireless visual sensor system for shiitake mushroom sorting. The visual detection was realized via the collection of images and cooperative transmission with the aid of visual sensors and Wi-Fi modules, respectively. So far, few relevant studies in this field have combined deep learning models with wireless visual sensors to solve mushroom-sorting issues. The model training results, the performance in the shiitake mushroom sorting, and the experiment results’ reliability were analyzed and discussed in detail and comprehensively.

The training results of the ViT model showed the efficiency of the model training. The performance in the shiitake mushroom sorting reflected the ViT model’s superiority compared to CNN. The developed system could classify shiitake mushrooms in real time with a stable accuracy rate, which ultimately will improve the economic benefits to the shiitake mushroom industry.

The deep-learning-based wireless sensor system for shiitake mushroom sorting can also be used to classify more crops with apparent visual features to reduce labor costs in the industry and improve production efficiency. In addition, it is also possible to combine binocular vision and multisensor technology with the current system to deal with classification work that requires a higher accuracy and minor feature identification.

## Figures and Tables

**Table 1:** Performance in ViT training.

Method	ViT-Tiny/16	ViT-Small/16	ViT-Small/32	ViT-Base/16	ViT-Base/32
Acc (%)	98.3	98.9	97.2	99.2	96.2
Train loss	0.401	0.361	0.391	0.317	0.383
Validation loss	0.127	0.118	0.128	0.057	0.137
Training time	115 s	177 s	139 s	472 s	271 s

**Table 2:** Sorting results of ViT.

Type	Acc (%)	PPV	TPR	F1-Score
Type A	98.53	1	0.9706	0.9851
Type B		0.9714	1	0.9855
Type C		0.9853	0.9853	0.9853

**Table 3:** Performance of ViT compared to CNN networks.

Methods	Params	Inference Time/Image	Train Time/Epoch	Acc (%)	Validation Loss
Resnet 50	24 M	13.2 ms	4.3 s	87.0	0.924
Inception v3	22 M	12.8 ms	3.6 s	96.4	0.166
Densenet 121	7 M	18.2 ms	3.6 s	93.8	0.225
SE-ResNeXt 50	26 M	12.5 ms	5.3 s	96.7	0.181
ViT-Tiny/16	6 M	6.8 ms	2.8 s	98.3	0.127
ViT-Base/16	86 M	8.7 ms	11.5 s	99.2	0.057

## Data Availability

Not applicable.
